# Why Living Kidney Donor Candidates Are Turned Down? A Single-Center Cohort Study

**DOI:** 10.7759/cureus.9877

**Published:** 2020-08-19

**Authors:** Abdulrahman Altheaby, Ahmed Almukhlifi, Abdulrahman Aldoukhi, Abdullah Alfaleh, Ghaleb Aboalsamah, Ala Alshareef, Mohamed Alruwaymi, Khaled Bin saad, Ziad Arabi

**Affiliations:** 1 Organ Transplant Center and Hepatobiliary Sciences Department, King Abdulaziz Medical City, Riyadh, SAU; 2 Medicine, King Saud Bin Abdulaziz University for Health Sciences, Riyadh, SAU; 3 Organ Transplant Center and Hepatobiliary Sciences Department, King Abulaziz Medical City, Riyadh, SAU

**Keywords:** living donor, donor evaluation, donor rejection, kidney transplant, transplantation, donor nephrectomy

## Abstract

Introduction

Living donor kidney transplantation is the best replacement therapy for patients with end-stage renal disease. It offers more benefits than deceased donor transplantation. However, living kidney donors (LKDs) undergo an extensive evaluation to ensure their suitability for donation, and this can result in rejection of many potential donors.

Aim

The aim of this study was to recognize the reasons for declining LKDs in our Organ Transplant Center at King Abdulaziz Medical City.

Settings and Design

This was a retrospective study to determine the various reasons to reject an LKD at the Organ Transplant Center.

Methods and Material

All the LKDs from January 2016 to December 2019 were included. Declined donors were reviewed and data were obtained from the electronic database and transplant nephrology shared files.

Statistical analysis

We performed data analysis using SPSS version 24.0 (IBM Corp., Armonk, NY, USA). Data for continuous variables were presented as mean ± standard deviation and were compared using t-test. Categorical variables were presented as frequencies and percentages; chi-square test was used to test for main association and then Bonferroni adjustment was used for post-hoc testing. Statistical significance was considered if a two-tailed p-value of <0.05 was achieved.

Results

A total of 410 potential LKDs were evaluated, of whom 241 (58.8%) successfully underwent donor nephrectomy and 169 (41.2%) were unable to proceed for kidney donation. The most common reasons for rejection of LKDs were medical (47.9%) followed by immunological reasons mainly blood group incompatibility (19.5%). Other reasons were donor withdrawal (15.4%), recipient-related reasons (7.1%), surgically unfit to proceed for nephrectomy (4.7%), or psychological reasons (2.3%).

Conclusions

A significant proportion of potential LKDs did not complete the kidney donation process due to medical, immunological, and surgical reasons. In addition, a proportion of LKDs decided to withdraw at some point during the evaluation process. Investing in donors’ educational programs and implementing a standardized evaluation process are essential to increase LKDs pool.

## Introduction

Renal transplantation is the best treatment option for patients with end-stage renal disease as it significantly improves the patients’ quality of life [1,2]. Kidney transplantation is typically classified as deceased-donor (brain death or cadaveric) or living-donor transplantation, which can be either from related or non-related donors, depending on the existence of a biological relationship between the donor and recipient. Renal transplantation from living donors offers more benefits than deceased donor transplantation since it is associated with longer graft survival and decreased waiting period on the transplantation list [3]. From 1979 to 2017, a total of 11,509 kidney transplantations were performed in Saudi Arabia, out of which, 7,838 (68.1%) came from living-related donors, 3,108 (27%) from deceased donors, and 563 (4.9%) were from living unrelated donors [4]. However, the number of kidney transplants per year only covers less than 5% of the dialysis population.

Potential living kidney donors (LKDs) undergo an extensive evaluation to ensure the short- and long-term safety before proceeding for donation [5]. All LKDs must be at least 18 years and have a body mass index (BMI) of less than 35 kg/m2, as well as good physical and mental health. In addition, donors should be free from uncontrolled hypertension, diabetes, active malignancy, or active infections [6]. All live kidney donation must be completely a voluntary decision without any pressure or guilt associated with the donation and donors cannot get paid for their donation [7].

By the end, accepting or declining LKDs should depend on whether the benefit outweighs the risk in both the donor and recipient or not. However, there are still considerable variabilities between transplant centers regarding acceptance or rejection of LKDs. Living donors' rejections can be due to medical, surgical, immunological, and/or psychological reasons. In this study, we report our experience with potential LKDs at King Abdulaziz Medical City (KAMC), Riyadh, Saudi Arabia. The main goal is to identify the reasons for declining potential LKDs in our Organ Transplant Center.

## Materials and methods

All rejected potential LKDs from January 2016 to December 2019 were reviewed and analyzed at the Organ Transplant Center at KAMC. Demographic data, recipient relation, evaluation process, and outcomes (accepted, rejected, withdrawn) were collected using electronic database retrospectively.

**Figure 1:**
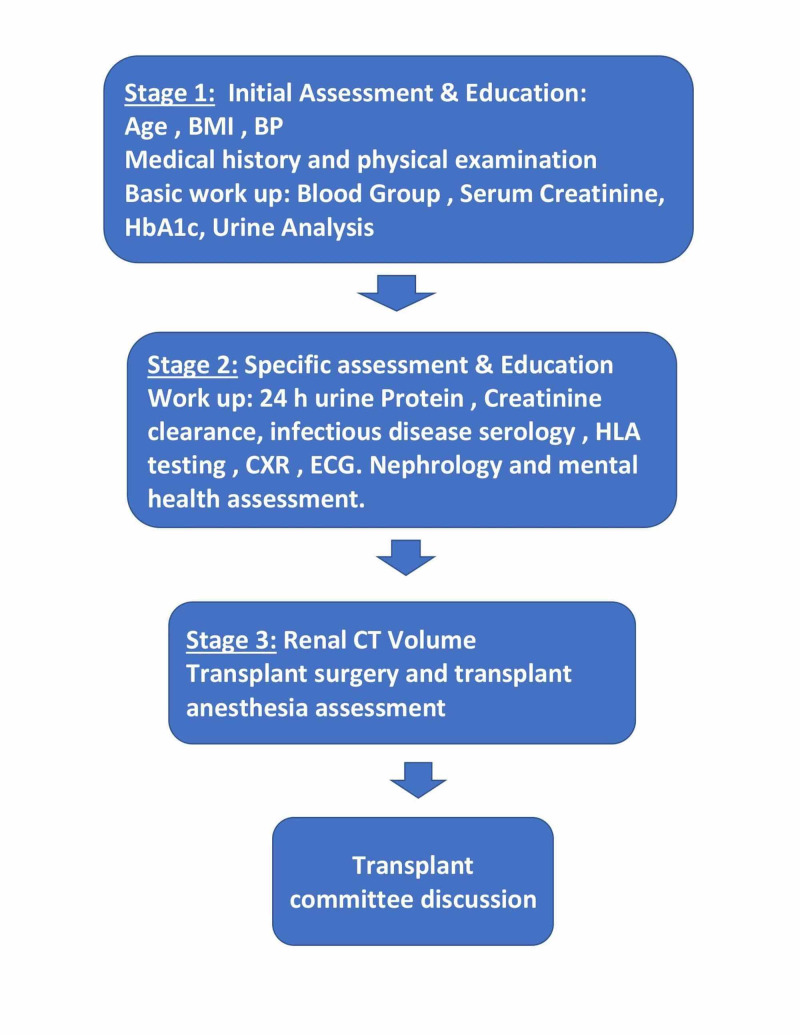
Three stages of kidney donors work-up BMI, body mass index, BP, blood pressure; HLA, human leukocyte antigen; CXR, chest X-ray; CT, computed tomography; ECG, electrocardiogram

The potential LKDs passed through stages of evaluation before being accepted for donor nephrectomy (Figure 1). In the first stage, potential LKDs meet the clinical transplant coordinator, who is responsible for assessing clinical basic and demographic data, as described, as well as taking a detailed history regarding metabolic, cardiac, infectious and renal diseases, and family and psychosocial history. Besides, the coordinator also revises the basic work-up that includes blood group, urine analysis, serum creatinine, and hemoglobin A1c (HbA1c). If the LKD is found to be a drug user, if the decision for donation is made under pressure, or if there is any payment involved with the donation, the LKD is rejected. Otherwise, if there are no contraindications, the evaluation process evolves to the second stage. At this stage, the LKD is submitted to further testing, including 24-hour creatinine clearance, viral serologies (such as HIV), human leukocyte antigens (HLA) tissue typing to perform virtual cross-match against the potential recipient. Subsequently, the potential donor would be interviewed by a nephrologist that reviews, once again, all the work-up, and the LKD is also assessed by a mental health specialist. If there are no clinical contraindications at this stage, the LKD is submitted to a renal CT angiogram (third stage) and evaluated by a transplant surgeon that presents the case to the Transplant Committee for final approval. The LKD may withdraw at any stage of work-up and the reason for that would be confidential.

Data analysis was performed using SPSS version 24.0 (IBM Corp., Armonk, NY, USA). Data for continuous variables were presented as mean ± standard deviation and were compared using t-test. Categorical variables were presented as frequencies and percentages; chi-square test was used to test for main association and then Bonferroni adjustment was used for post-hoc testing. Statistical significance was considered if a two-tailed p-value of <0.05 was achieved.

## Results

A total of 410 potential LKDs were evaluated at the KAMC Organ Transplant Center from January 2016 to December 2019. Of those, 241 (58.8%) successfully underwent donor nephrectomy, whereas 169 (41.2%) were unable to proceed for kidney donation due to different reasons. There was no specific pattern regarding the annual rate of LKDs rejection during the study period, as shown in Table 1.

**Table 1:** Annual rate of donors revaluation (accepted or declined)

Year	Accepted, N (%)	Declined, N (%)
2016	43 (52)	37 (48)
2017	47 (53.3)	36 (46.7)
2018	69 (63.9)	39 (36.1)
2019	96 (62.8)	57 (37.2)

There was no significant difference in accepted and rejected donors’ demographics in terms of average age (29 years in accepted vs. 31 years in rejected group) and male predominance (79.6% in accepted vs. 86.3% in rejected donors). More than half of the donors were non-smokers in both groups (59.3% of the accepted group and 54.4% of the rejected group). Most of LKDs’ BMI was between 18.5 and 29.9 kg/m^2^ in both groups, although more patients had a BMI of >30 kg/m^2^ in the rejected group versus donated candidates (32% vs. 13.7%). Regarding kidney function, similar average glomerular filtration rate (GFR) was found in both accepted (113mL/min/1.73 m^2^) and rejected donors (111 mL/min/1.73 m^2^), and similar average creatinine (mg/dL) was found and in accepted (71.6 mg/dL) and rejected donors (73.6 mg/dL). HbA1c was also similar, with 5.1 to 5.3 in both, groups and majority of candidates were non-hypertensive in both groups. However, hypertension was more prevalent among rejected LKDs. Table 2 showed the details of demographic data of all evaluated LKDs.

**Table 2:** Demographic data of potential LKDs eGFR, estimated glomerular filtration rate

		Successfully completed the donation process	Did not the complete donation process	p-Value
Total, n = 410		241 (58.8%)	169 (41.2%)	
Age (years)		31.3 ± 8.5	32.2 ± 8.06	0.281
Gender	Male	192 (79.7%)	126 (74.6%)	0.221
	Female	49 (20.3%)	43 (25.4%)	
Hypertension	Yes	3 (1.2%)	12 (7.1%)	0.001
	No	238 (98.8%)	157 (92.9%)	
Smoker	Yes	98 (40.7%)	77 (45.6%)	0.323
	No	143 (59.3%)	92 (54.4%)	
BMI (kg/m^2^)	<18.5	12 (5%)	7 (4.1%)	0.104
	18.5-24.9	86 (35.7%)	52 (30.7%)	
	25-29.9	110 (45.6%)	71 (42%)	
	> 30	33 (13.7%)	39 (23%)	
Blood group	O	146 (60.6%)	123 (72.8%)	0.045
	A	44 (18.3%)	26 (15.4%)	
	B	48 (19.9%)	18 (10.7%)	
	AB	3 (1.2%)	2 (1.1%)	
Creatinine (μmol/L)		71.62 ± 10.61	73.69 ± 25.92	0.265
eGFR (mL/min/1.73 m^2^)		113 ± 23.34	111.06 ± 34.12	0.494
HbA1c		5.25 ± 1.78	5.14 ± 2.03	0.561

**Figure 2:**
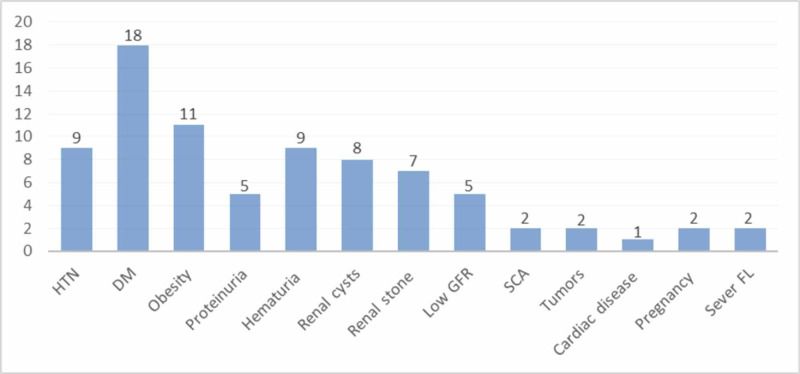
Medical reasons for declining potential living kidney donors HTN, hypertension, DM, diabetes mellitus; GFR, glomerular filtration rate; SCA, sickle cell anemia; Sever FL, severe fatty liver

Table 3 demonstrates the relationship of potential donors with the recipients. Most of the donors were related to the recipients, with 192 (79.7%) in donated LKDs and 110 (65%) in rejected LKDs. There were more unrelated donors in the rejected group than those who proceeded to donor nephrectomy (59 [35%] vs. 49 [20.3%], respectively). The reasons for declining potential LKDs were classified into several subclasses, as shown in Table 4. The most common reason to decline potential LKDs was the presence of medical reasons in 81 (47.9%) out of 169 rejected LKDs. Medical reasons were divided into several causes outlined in Table 5 and Figure 2. Diabetes mellitus or impaired glucose tolerance, hypertension, and obesity represented almost 50% of the medical reasons for rejecting LKDs. The second reason for declining potential donors was HLA or ABO incompatibility, with 33 patients corresponding to 19.5% of the rejected potential LKDs. Rejection due to surgical reasons such as renal vascular anomalies or double ureter was found in 8 (4.7%) LKDs. Four LKDs were rejected after mental assessment (2.3% of the rejected LKDs). A total of 26 (15.4%) potential LKDs decided to withdraw their consent to donate, of whom 17 (65%) LKDs were unrelated to recipient. While 12 (7.1%) LKDs were willing to proceed for kidney donation and appeared good candidates, but they were rejected for recipients' related reasons. In our cohort, there were five (2.9%) LKDs who were rejected for unknown reasons; we could not identify the reasons, but it seems due to recipient-related issues because their work-up was fine to proceed for donation.

**Table 3:** The relationship of potential donor with the recipient ^a^Significant difference was found when column proportions were compared after p-value adjustment using the Bonferroni method.

Relation to recipient	Successfully completed donation process, N (%)	Did not complete donation, N (%)	p-Value
Parent	8 (3.3%)	6 (3.6%)	0.026
Offspring	84 (34.9%)^a^	32 (18.9%)^a^	
Sibling	52 (21.6%)	39 (23.1 %)	
Spouse	12 (5%)	11 (6.5 %)	
Uncle/aunt	11 (4.6%)	8 (4.7 %)	
Cousin	19 (7.9%)	19 (11.2 %)	
Nephew/niece	6 (2.5%)	2 (1.2%)	
Unrelated	49 (20.3%)^a^	52 (30.8%)^a^	

**Table 4:** Reasons for declining potential living kidney donors (n = 169)

Reason	Number (%)
Medical	81 (47.9%)
Immunologic	33 (19.5%)
Surgical	8 (4.7%)
Recipient related	12 (7.1%)
Psychological	4 (2.3%)
Back out	26 (15.4%)
Unknown	5 (2.9%)

**Table 5:** Medical reasons for declining potential living kidney donors SCA, sickle cell anemia; FL, fatty liver

Reason	N (%) = 81 (47.9)
Hypertension	9
Diabetes mellitus or impaired glucose tolerance	18
Obesity	11
Proteinuria	5
Hematuria	9
Renal cysts	8
Renal stone	7
Low glomerular filtration rate	5
SCA/thalassemia	2
Tumors	2
Cardiac disease	1
Pregnancy	2
Severe FL	2

## Discussion

Living kidney donation contributes to more than 80% of kidney transplantation in Saudi Arabia [4]. The low rate of deceased donor kidney transplants in Saudi Arabia occurs due to many reasons, of which the most important is related to the cultural background [8]. For this reason, it is essential to expand the living donor pool to meet the rapidly increasing need for organ transplantation in Saudi Arabia. This should be done carefully to ensure living donors and recipient safety. Understating our current practice in LKDs evaluation is very essential to set a protocol to safely expand the LKDs pool.

In our study, more than half (58.8%) of the evaluated potential LKDs successfully underwent donor nephrectomy, which was similar to another study conducted in New York in which donor nephrectomy was proceeded in (56%) of their fully evaluated kidney donors [9]. This percentage of donors proceeding for donor nephrectomy was higher when compared to a local study conducted at King Fahad Specialist Hospital (KFSH) in Dammam and another study in Turkey in which donor nephrectomy was proceeded only in 32% and 38% of their evaluated donors, respectively [10,11].

The majority of the potential donors in our study were young with no significant difference in average age between accepted (29 years) and rejected (31 years) donors, which was similar to the KFSH study where the average age of potential LKDs was 32 years [10]. In contrast, the potential LKDs in our study were younger than LKDs of other studies from western countries where the average age was between 45 to 53 years [12,13]. Also, our study showed that both groups were predominantly males (79.6% vs. 86.3%). This finding was similar to the KFSH study with a ratio of 3:1 (M: F), which could be explained by the cultural background where males usually take the initiative in helping the community [10]. This finding was the opposite to what was found in other studies where female gender predominated [13,14].

Our study also demonstrated that more than half of the donors were non-smokers in both groups (59.3% and 54.4%). In general, we advised our donors to stop smoking and offer medical and psychological support to help them quit smoking but we did not decline a donor who is a smoker. In a risk index lately established for LKDs, a history of smoking was a crucial factor in the living donor in comparison to each other and to deceased donor kidneys [15]. Furthermore, the history of cigarette smoking by donors was statistically an important risk factor for both graft and survival of recipients [16].

Regarding the reasons for the rejection of potential LKDs, the most common were medical reasons (47.9%). Of these, diabetes or impaired glucose tolerance were the most frequent followed by obesity and hypertension. Similarly, other local and global studies showed that the most common reasons for the rejection of potential LKDs were medical with diabetes and hypertension constituting the majority of the medical reasons [10,11,17]. At our center, we decline all potential LKDs with diabetes or impaired glucose tolerance based on fasting blood glucose and HbA1c. Furthermore, most of LKDs’ BMI was between 18.5 and 29.9 kg/m^2^ in both groups, although more patients had a BMI of >30 kg/m^2^ in the rejected group versus donated candidates (32% vs. 13.7%). In our program, we accept only LKDs with a BMI of <30 kg/m^2^. Donors with a BMI of >30 kg/m^2^ are counselled to lose weight and encouraged to contact the transplant coordinators when they reach their target weight. Acceptable BMI cutoffs are variable between transplant centers. A survey was conducted by Mandelbrot et al. in 2007 who reported that 50% of transplant programs use a 35 kg/m^2^ limit in the body mass index [18]. Guidelines for KDIGO (Kidney Disease: Improving Global Outcomes) 2017 propose that the decision to accept donor candidates with BMI > 30 kg/m^2^ should be taken on a case-by-case basis considering medical conditions and demographic factors [19]. Majority of candidates in our study were non-hypertensive in both groups. Normal blood pressure is appropriate for donation as specified by guidelines for the general population in the country or area where a donation is scheduled. Donor candidates with hypertension that can be controlled with one or two antihypertensive agents to less than 140/90 mmHg and have no evidence of target organ damage could be acceptable for donation [20].

Kidney function evaluation is essential pre-donation. Kidney donors should have normal renal function, without proteinuria or hematuria. In our study, only 5% LKDs were rejected because of low GFR. In our practice, we follow KDIGO 2012 Chronic Kidney Disease (CKD) guidelines for renal function evaluations [21,22]. GFR of 90 mL/min/1.73 m^2^ or greater should be considered an appropriate degree of kidney function for kidney donation, whereas donor candidates with GFR less than 70 mL/min/1.73 m^2^ should no longer donate. LKDs with GFR between 70 and 90 mL/min would be individualized based on donor age, comorbidity, and other factors. In our study, we found that both groups had similar renal function estimation with almost similar average GFR (mL/min/1.73 m^2^) of 113 and 111.06, which was different from an Australian study where 15% of accepted candidates were found to have a GFR of less than 80 mL/min/1.73 m^2^ [23]. This could be explained by the younger age in our cohort. Overt proteinuria is a contraindication for living donation, whereas persistent microalbuminuria is considered a high risk for donation [24]. In our study, only 5% of potential LKDs were rejected because of persistent proteinuria and 7% were rejected due to persistent microscopic hematuria. In general, donor candidates with persistent microscopic hematuria defined as the presence of >3 RBC/hpf should have thorough urological evaluation and consideration of a kidney biopsy prior to donation. Persistent hematuria of glomerular origin is a contraindication to living donation because it may indicate renal disease in the donor. However, donors with thin basement membrane disease might be considered in select cases [25]. In our study, kidney biopsies were performed for three donors, of whom one was found to have Alport disease and the other two had IgA (immunoglobulin A) nephropathy. Practices in evaluating and accepting prospective donors with a history of nephrolithiasis vary across transplant centers [26,18]. At our center, prospective donors with a history of recurrent unilateral renal stone, bilateral renal stones, or positive metabolic predisposition to stones formation were not accepted for donation. In our study, 7 (8.6%) potential LKDs were rejected because of nephrolithiasis.

ABO, HLA, and Rh incompatibility were the second most common reasons for rejection in 19.5% of the potential LKDs. This is actually contrary to a study conducted in India that showed that blood group incompatibility was the most common reason for rejection (45.8%) followed by diabetes, renal diseases, and hypertension [27]. In our center, we adopted desensitization protocols, and we are in the process to start paired kidney exchange program to minimize donor rejections due to immunological barriers [28].

Declining LKD because of surgical reasons accounted for only 4.7% of declined donors, although reports were varied from transplant centers. Connaughton et al. found that 13% of donors declined due to surgical contraindications [12]. The KFSH study showed that 7.9% of donor rejected for surgical reasons [10]. The main surgical reason to decline an LKD was having complex vasculature, making them unsuitable candidates.

In our study, 15.3% of evaluated LKDs withdrew their consent to donate at different stages of work-up. This was considered a high percentage as compared to a study conducted in Italy showing that 6.3% of the reasons of not to proceed for donor nephrectomy were due to donor withdrawal [14]. Almost 30% of these donors in our study withdrew their consent after completing work-up, which led to a significant waste of resources. Our analysis showed that 17 out of 26 donors who withdrew were unrelated to recipients, although they completed two meetings with the ethical committee for living unrelated donors. This was in agreement with a multivariate analysis by Bailey et al. showed that friend donors were more likely to withdraw from donation [29]. In our cohort, we could not identify clear reasons for donor withdrawal because this information was confidential and not documented in donor records. Studying the reasons for potential LKD withdrawal would allow the transplant programs to improve educational programs and evaluation process to improve living donor recruitment and retention.

This study has several limitations. This is a retrospective, single-center study. The data for this study were obtained by chart review and it was difficult to obtain detailed information that might have influenced the decision of accepting or rejecting the donation. However, despite these limitations, this study reflects our current practice and we need to design a better system to recruit and retain LKDs in an efficient and safe way.

## Conclusions

In conclusion, there is a significant number of potential LKDs that did not proceed for kidney donation. There were different reasons such as medical, immunological, surgical, and consent withdrawal. Working on measures to minimize rejection is essential to meet the transplantation demands. Accepting more marginal donors such as well-controlled hypertension, BMI 30-35 kg/m^2^ without other cardiovascular risk factors, adopting paired exchange programs at a national level in Saudi Arabia, and expanding desensitization protocols to overcome immunologic barriers are possible pathways to improve the transplantation rates. In addition, providing more education for LKDs who withdraw and give them more time to come back for donation could increase the LKD pool.
